# Cancer survival in New South Wales, Australia: socioeconomic disparities remain despite overall improvements

**DOI:** 10.1186/s12885-016-2065-z

**Published:** 2016-02-01

**Authors:** Julia F. Stanbury, Peter D. Baade, Yan Yu, Xue Qin Yu

**Affiliations:** Sydney School of Public Health, The University of Sydney, Sydney, Australia; Cancer Research Division, Cancer Council New South Wales, P.O. Box 572, Kings Cross, NSW 1340 Australia; Cancer Research Centre, Cancer Council Queensland, Brisbane, Australia; School of Public Health and Social Work, Queensland University of Technology, Brisbane, Australia

**Keywords:** Cancer, Survival analysis, Socioeconomic variation, Disparity

## Abstract

**Background:**

Disparities in cancer survival by socioeconomic status have been reported previously in Australia. We investigated whether those disparities have changed over time.

**Methods:**

We used population-based cancer registry data for 377,493 patients diagnosed with one of 10 major cancers in New South Wales (NSW), Australia. Patients were assigned to an area-based measure of socioeconomic status. Five-year relative survival was estimated for each socioeconomic quintile in each ‘at risk’ period (1996–2000 and 2004–2008) for the 10 individual cancers. Poisson-regression modelling was used to adjust for several prognostic factors. The relative excess risk of death by socioeconomic quintile derived from this modelling was compared over time.

**Results:**

Although survival increased over time for most individual cancers, Poisson-regression models indicated that socioeconomic disparities continued to exist in the recent period. Significant socioeconomic disparities were observed for stomach, colorectal, liver, lung, breast and prostate cancer in 1996–2000 and remained so for 2004–2008, while significant disparities emerged for cervical and uterus cancer in 2004–2008 (although the interaction between period and socioeconomic status was not significant). About 13.4 % of deaths attributable to a diagnosis of cancer could have been postponed if this socioeconomic disparity was eliminated.

**Conclusion:**

While recent health and social policies in NSW have accompanied an increase in cancer survival overall, they have not been associated with a reduction in socioeconomic inequalities.

**Electronic supplementary material:**

The online version of this article (doi:10.1186/s12885-016-2065-z) contains supplementary material, which is available to authorized users.

## Background

Internationally, cancer patients from more socioeconomically disadvantaged backgrounds have been shown to have poorer outcomes for many major cancers [1–4]. Similar socioeconomic disparities in survival have also been reported in Australia [5, 6]. In the few studies that have monitored such disparities over time in a population, most report either no change in the extent of disparities detected or widening disparities, for several major cancers [7–9]. Generally these studies report on only one or few cancer types and involve limited adjustment for potential prognostic factors.

In 2008, Yu et al reported that persons from more socioeconomically disadvantaged areas of New South Wales (NSW), Australia experienced poorer survival for many types of cancer than those from the least disadvantaged areas [6]. These disparities are well recognised by health professionals and providers; however there is little knowledge about whether these socioeconomic disparities in cancer survival have reduced over time.

The purpose of this study is to determine whether the socioeconomic variations in cancer survival for 10 major cancers in NSW, Australia have changed over time, after account for the impact of demographics and tumour characteristics.

## Methods

Data were obtained from the population-based NSW Central Cancer Registry for all patients aged 15–89 years at the time of their diagnosis of a primary cancer between January 1991 and December 2008. Notification of cancer diagnosis to the registry is a statutory requirement in NSW. We included ten cancers with high incidence and large contribution to mortality (see Table 1), defined by International Classification of Diseases for Oncology 3^rd^ Edition codes [10].

**Table 1:** Five-year relative survival (%) by socioeconomic disadvantage for 10 cancers in NSW, Australia, 1996–2000 and 2004–2008

Cancer	Five-year relative survival (%)									
	1996–2000					2004–2008				
	Least	Second	Third	Fourth	Most	Least	Second	Third	Fourth	Most
Stomach (C16)	33.4	24.6	27.6	24.9	25.2	35.2	31.6	28.1	31.0	26.1
Colorectum (C18-21)	63.5	60.8	60.9	59.6	60.2	68.7	66.0	66.1	64.6	64.7
Liver (C22)	22.7	16.3	10.6	13.4	11.5	22.6	18.4	14.7	19.8	17.1
Lung (C33-34)	16.2	16.4	14.5	15.0	14.5	18.1	17.2	16.3	17.0	14.2
Melanoma (C43)	91.0	91.1	92.2	88.8	90.2	92.1	90.9	90.3	89.2	90.4
Breast (C50)	87.8	85.7	83.9	83.9	83.2	92.6	88.5	87.9	88.4	89.2
Cervix (C53)	71.7	72.9	74.3	69.0	73.2	76.2	72.0	75.5	73.4	60.9
Uterus (C54-55)	81.4	80.5	79.0	80.0	79.2	84.8	77.2	74.5	79.1	83.1
Ovary (C56-57)	44.4	41.6	38.5	38.9	38.2	44.5	44.5	44.7	39.4	41.0
Prostate (C61)	85.9	84.0	84.5	83.4	81.8	94.4	93.6	91.0	94.5	90.6

Cases were followed up for survival status up to the 31 December 2008 through record linkage of the cancer cases in the Cancer Registry with death records from the NSW Register of Births, Deaths and Marriages and the National Death Index. Cases notified to the registry by death certificate only or first identified at post-mortem were excluded.

To maintain comparability with the previously mentioned study by Yu and colleagues [6], we used an area-based socioeconomic measure, the “Index of Education and Occupation” score. This is a composite index of relative advantage, based on data from the national Australian census [11]. Index scores derived from the 2001 census were used to classify the included cases by socioeconomic status (SES) in both analysis periods. An area with a high index score indicates a relatively high level of educational attainment and skilled employment of the resident population. Socioeconomic quintiles were created by ranking the index score of all the Local Government Areas (LGA) in NSW. In 2001 there were 175 LGAs in NSW, ranging from small urban areas with large populations to extremely large rural areas with small populations, each with an average population of 35,954 residents (IQR: 4713–43,809) [ABS Online data 2001]. Cases were excluded from analysis if they had insufficient information to assign an LGA or if index scores were not available.

Disease stage at diagnosis was based on pathology reports and statutory notifications by hospitals, then coded using a modified summary classification: localised (stage I), regional (a combination of stages II and III), distant (stage IV) and unknown (including missing) stage.

### Statistical Analysis

Relative survival, the ratio of the observed proportion surviving in a group of cancer patients to the expected proportion that would have survived in an age- and sex-comparable group of people from the general population [12], was used in this analysis because we used all-cause mortality from a population-based cancer registry. Survival time for each case was calculated from the month of diagnosis to the month of death or censoring (31 December 2008) using life-table methods [13]. Expected survival was calculated using the Pohar-Perme method [14]. We constructed SES-specific life tables for each year 1996–2000 and 2004–2008 by collapsing all-cause mortality data and corresponding population data by LGA into the SES quintiles used for classifying cancer cases. The period method [15] was used as in the previous study [6]. For each of these two ‘at risk’ periods (1996–2000 and 2004–2008), we calculated 5-year relative survival by SES quintile for 10 individual cancers. We chose the two ‘at risk’ periods for analysis to allow a reasonable “lead in time” from the start of the diagnostic cohort (1991) and to enable sufficient time for changes in survival disparity to occur.

We investigated the effect of SES on survival for each cancer using multivariate modelling to adjust for potentially confounding variables. Firstly, we calculated the relative excess risk (RER) of death due to cancer using a Poisson-regression model [16]. In this model, the main-effect variables were SES quintile, age group at diagnosis (<49 years, 50–59 years, 60–69 years, 70–79 years, 80–89 years), sex, year of follow-up (1–5 years) and cancer stage at diagnosis. We included the natural logarithm of the population size as the offset variable. The RER derived from this model is the ratio of the excess risk of death in a given SES quintile to the reference SES group (the least disadvantaged quintile) after controlling for the other factors included in the model. Ninety-five percent confidence intervals (CIs) for the RERs were calculated using the estimated coefficients and standard errors from the Poisson model. Secondly, we added an interaction term between SES quintile and time period to the model, to allow the effect of SES to change between periods and then used a likelihood ratio test between the nested models to determine if this interaction was significant.

Finally, an estimate of the number of lives potentially extendable to 5 years from cancer diagnosis was calculated for the four more disadvantaged SES quintiles for each period. This was done in three steps. First, for each of the four disadvantaged quintiles, we calculated the difference between the number of stage-adjusted deaths within a specific cancer cohort and that of an age-sex equivalent group in the general population of the same quintile [17]. These are the observed number of excess deaths. Second, we calculated the number of deaths that would have occurred if the stage-adjusted RER of cancer death for these quintiles equalled that of the least disadvantaged quintile at 5 years from diagnosis [6]. These are the optimum number of excess deaths. The number of potentially extendable lives is equal to the difference between the observed number of excess deaths and the optimum number of excess deaths. This measure, similar to that used in the EUROCARE-4 study [18], among others [19, 20], has been used in different health settings and is exchangeable with “avoidable deaths” and the “number potentially saved” within a set time period since diagnosis. A Pearson chi-square test was then used to determine if the two proportions of “extendable” lives were significantly different over time.

All significance tests with *p*-value <*0.05* were taken to indicate statistical significance. Statistical analyses were completed using STATA software, v13.1 (StataCorp LP: College Station, TX).

## Results

A total of 380,306 cases diagnosed between 1991 and 2008 that were prevalent cases between periods of 1996–2000 and 2004–2008 were identified. About 0.7 % (2 663 cases) were excluded from analysis due to being notified to the registry by death certificate only or first identified at post-mortem, while a further 150 cases were excluded due to missing SES data. In total, 139,234 cases at-risk in 1996–2000 and 238,259 cases at-risk in 2004–2008 were included in the final cohort (online Additional file 1: Table S1). The numbers of cases included in the analysis increased over time and were relatively evenly distributed across the socioeconomic quintiles in both periods. Liver, breast, ovarian and prostate cancers saw higher case numbers in the less disadvantaged SES groups, whereas the opposite trend occurred for lung cancer.

Relative survival increased over time for the majority of cancers, as shown in Table 1. However, the socioeconomic disparities observed in the first period (1996–2000) remain broadly similar in the late period (2004–2008).

**Fig. 1:**
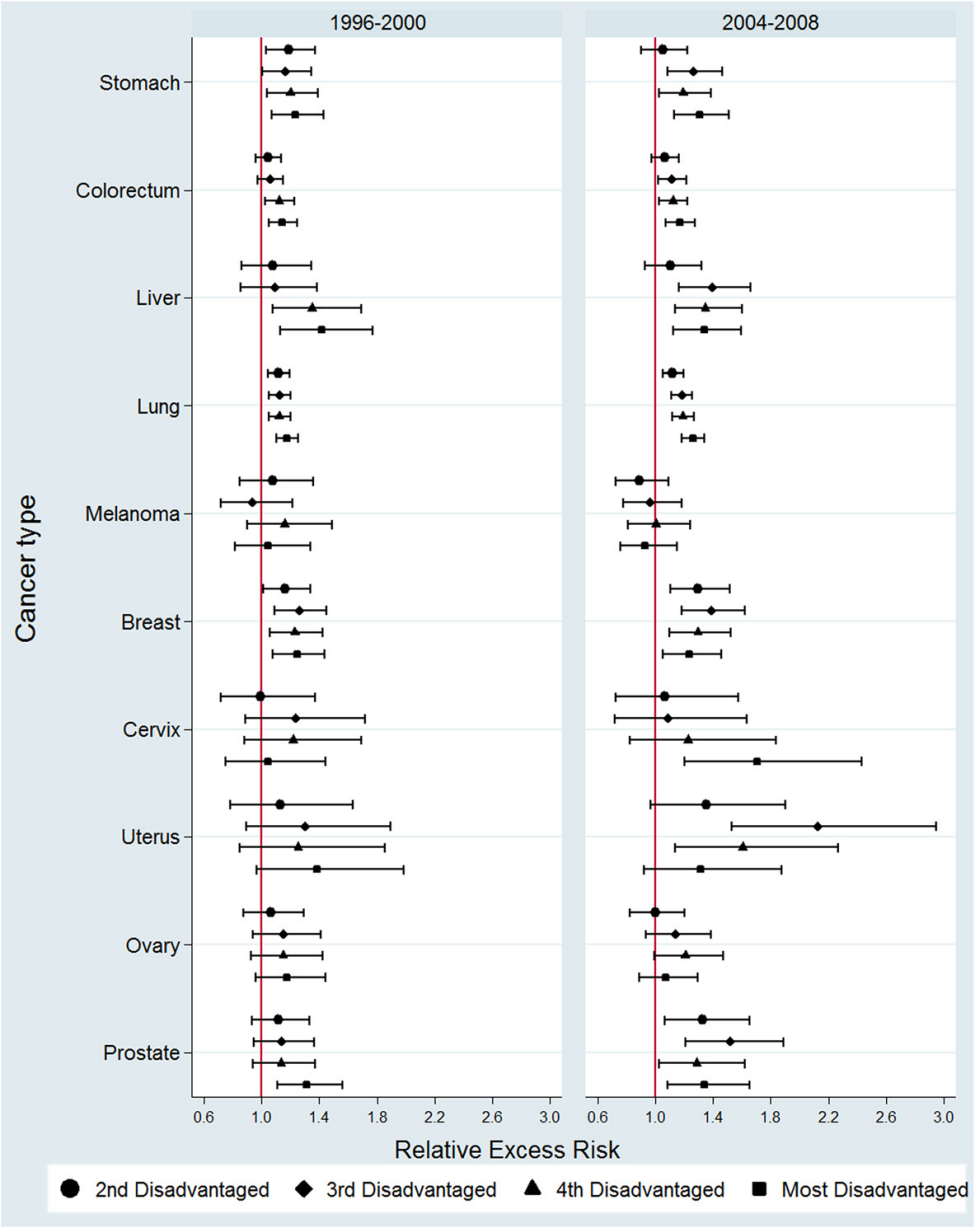
Relative excess risk* by socioeconomic disadvantage for 10 cancers in New South Wales, Australia, 1996–2000 and 2004–2008. ^*^The RER of the reference group (least disadvantaged SES quintile) was set to 1.00

Figure 1 shows the results of the multivariable modelling: RERs by SES quintile (with the reference group being the least disadvantaged quintile). Values of these RER estimates and *p*-value of significance tests are presented in Table 2. During 1996-2000, the RER of death was significantly higher for more disadvantaged patients with stomach, colorectal, liver, lung, breast and prostate cancers. No significant variation in RER was found for melanoma, ovarian, cervix or uterine cancers. By the period of 2004–2008, significant RER’s continued to exist for, stomach, colorectal, liver, lung, breast, prostate cancers, while RER variations in cervical and uterine cancers became highly significant (*p* = *0.008* and *0.001* respectively). Melanoma and ovarian cancer again showed no significant variation in RER of death by SES in 2004–2008.

**Table 2:** Relative excess risk of death by socioeconomic disadvantage for 10 cancers in NSW, Australia, 1996–2000 and 2004–2008

Cancer type	Relative excess risk of death^a^ and (95 % confidence interval)												
	1996–2000						2004–2008						*p-*value^c^
	Least	2nd	3rd	4th	Most	*p-value* ^b^	Least	2nd	3rd	4th	Most	*p-*value^b^	
Stomach	1.00	1.19 (1.03, 1.37)	1.16 (1.00, 1.34)	1.20 (1.04, 1.39)	1.24 (1.07, 1.43)	*0.04*	1.00	1.05 (0.91, 1.22)	1.26 (1.08, 1.46)	1.19 (1.02, 1.39)	1.31 (1.13, 1.51)	*0.001*	*0.40*
Colorectum	1.00	1.05 (0.96, 1.14)	1.06 (0.97, 1.15)	1.12 (1.03, 1.23)	1.15 (1.05, 1.25)	*0.01*	1.00	1.06 (0.98, 1.16)	1.11 (1.02, 1.22)	1.12 (1.03, 1.23)	1.17 (1.07, 1.27)	*0.005*	*0.93*
Liver	1.00	1.08 (0.86, 1.34)	1.09 (0.86, 1.39)	1.35 (1.08, 1.69)	1.42 (1.13, 1.77)	*0.008*	1.00	1.11 (0.93, 1.32)	1.39 (1.16, 1.66)	1.35 (1.13, 1.60)	1.34 (1.13, 1.60)	*0.0003*	*0.44*
Lung	1.00	1.12 (1.05, 1.19)	1.12 (1.05, 1.20)	1.12 (1.05, 1.20)	1.17 (1.10, 1.25)	*<0.0001*	1.00	1.12 (1.05, 1.19)	1.18 (1.11, 1.26)	1.19 (1.12, 1.27)	1.26 (1.19, 1.34)	*<0.0001*	*0.55*
Melanoma	1.00	1.08 (0.85, 1.36)	0.93 (0.72, 1.21)	1.16 (0.90, 1.49)	1.05 (0.82, 1.34)	*0.53*	1.00	0.89 (0.72, 1.09)	0.96 (0.78, 1.18)	1.00 (0.81, 1.24)	0.93 (0.76, 1.15)	*0.77*	*0.74*
Breast	1.00	1.16 (1.01, 1.34)	1.26 (1.09, 1.45)	1.23 (1.06, 1.43)	1.25 (1.08, 1.44)	*0.009*	1.00	1.30 (1.11, 1.51)	1.38 (1.18, 1.62)	1.29 (1.10, 1.52)	1.24 (1.05, 1.46)	*0.001*	*0.74*
Cervix	1.00	0.99 (0.72, 1.37)	1.24 (0.89, 1.72)	1.22 (0.88, 1.69)	1.04 (0.75, 1.44)	*0.52*	1.00	1.07 (0.72, 1.58)	1.09 (0.72, 1.63)	1.23 (0.82, 1.83)	1.71 (1.20, 2.43)	*0.008*	*0.11*
Uterus	1.00	1.13 (0.78, 1.63)	1.30 (0.90, 1.90)	1.25 (0.85, 1.86)	1.39 (0.97, 1.99)	*0.43*	1.00	1.35 (0.97, 1.90)	2.12 (1.53, 2.95)	1.61 (1.14, 2.27)	1.32 (0.92, 1.88)	*0.0001*	*0.21*
Ovary	1.00	1.07 (0.88, 1.29)	1.15 (0.94, 1.41)	1.15 (0.93, 1.42)	1.18 (0.96, 1.44)	*0.48*	1.00	1.00 (0.83, 1.20)	1.14 (0.93, 1.38)	1.21 (0.99, 1.47)	1.07 (0.89, 1.30)	*0.25*	*0.87*
Prostate	1.00	1.12 (0.94, 1.33)	1.14 (0.95, 1.36)	1.14 (0.94, 1.37)	1.32 (1.11, 1.56)	*0.03*	1.00	1.33 (1.07, 1.66)	1.51 (1.21, 1.89)	1.29 (1.02, 1.62)	1.34 (1.08, 1.66)	*0.008*	*0.35*

^a^Adjusted for age group, sex, year of follow-up and stage at diagnosis in a Poisson model

^b^Wald test for the effect of SES quintiles in the Poisson model

^c^Wald test for interaction between time period and SES quintiles

The total of excess deaths due to cancer in 1996–2000 was 25,420 for all 10 cancers, of which 2690 lives (10.6 % of excess deaths) were potentially extendable if the SES survival disparity did not exist (Table 3). The corresponding number for 2004–2008 increased to 26,583, of which 4253 lives (16.0 % of excess deaths) were potentially extendable. The increase in the proportion of extendable lives over time was significant (*p* < *0.001*) for the majority of cancers. Lung, colorectal and breast cancers respectively accounted for the greatest numbers of extendable lives in both periods.

**Table 3:** Number of lives that might be extended beyond 5 years from diagnosis for 10 cancers in NSW, Australia 1996–2000 and 2004–2008

Cancer	Number of lives potentially extended						*p-value* ^c^
	1996–2000			2004–2008			
	Number of excess deaths	Lives potentially extended^b^	Proportion of excess deaths (%)	Number of excess deaths	Lives potentially extended^b^	Proportion of excess deaths (%)	
Stomach	1967	316	16.1	1895	324	17.1	*0.389*
Colorectum	6069	443	7.3	6189	606	9.8	*<0.001*
Liver	739	137	18.6	1312	312	23.8	*0.006*
Lung	9729	1090	11.2	11,002	1779	16.2	*<0.001*
Melanoma	941	0^a^	0	1213	0^a^	0	
Breast	2188	393	17.9	1855	472	25.5	*<0.001*
Cervix	388	0^a^	0	318	92	28.9	*<0.001*
Uterus	356	0^a^	0	474	256	54.0	*<0.001*
Ovary	970	0^a^	0	1051	0^a^	0	
Prostate	2073	311	15.0	1275	411	32.3	*<0.001*
All of the above	25,420	2690	10.6	26,583	4253	16.0	*<0.001*

^a^RER coefficients are not significant in the relative survival model for specific cancer

^b^Estimated by equating the RER of death due to cancer in the four more disadvantaged SES quintiles to that of the least disadvantaged quintile and calculating the difference in number of cancer deaths

^c^Pearson chi-square test of the difference between proportions of excess deaths over time (two periods)

## Discussion

We found that while survival for 10 cancers has either remained stable or increased over time, patients living in more disadvantaged areas of NSW have continued to experience lower survival rates than the least disadvantaged patients for cancers of the stomach, colorectum, liver, lung, female breast and prostate and new disparities have emerged for cervical and uterine cancer.

There are several strengths in the design and methods of this study. Our population-based data reflect the survival experience of people diagnosed with major types of cancer in NSW Australia. We used a well-established ecological study design and statistical methods, as used previously and recommended for measuring socioeconomic inequalities in health [6, 11]. In addition, we provide two measures of socioeconomic disparity, one relative (RER) and one absolute (number of lives potentially extendable). The availability and adjustment for stage of disease at diagnosis data further strengthens our analysis, as stage is widely known to be an important predictor for cancer survival [21, 22].

A limitation of our study comes from the use of aggregated area-level data to classify patients according to SES. Individual level socioeconomic data for cancer patients was not available for this study. However, recent studies using individual-level socioeconomic data detected comparable trends in cancer survival disparities [20, 23], suggesting a similar impact of individual and area-based measures of SES on cancer survival. Area-level methods for measuring health disparities have been validated previously and were shown to appropriately detect trends in survival inequalities [24]. In addition, the index used in this study has been extensively reviewed and validated using nine different methods [11] and has been widely used as a socioeconomic measure in numerous studies of different health outcomes in Australia [6, 25, 26].

Previous research has shown that the definition of the socioeconomic index generally has little impact on the survival disparities detected [27]. Under Australia’s universal healthcare system, access to health care is (theoretically) independent of a patient’s financial resources. As such, compared to the index used here, other income-based or economic-disadvantage indicators of SES may be less relevant to identifying disparities in this context.

Our results of increased survival from cancer overall and continuing socioeconomic disparities in survival are consistent with both current Australian and international evidence. Persistent survival disparities by SES have been found for stomach [28], colorectal [29], liver [30], lung [1], breast [1, 21, 31], cervical [1, 29], uterine [29] and prostate [29, 31] cancers. The reasons for the socioeconomic survival disparities are not thoroughly understood, and evidence on contributing factors is both limited and often inconclusive. Some factors thought to contribute to survival disparities by SES relate to differences in diagnosis and treatment factors, patient characteristics and health care system features [2].

Previous studies of ovarian cancer survival have also found no association with SES [6, 32]. The non-specific nature of symptoms and lack of a definitive screening-diagnostic test could explain this finding, as the majority of diagnoses in all socioeconomic groups in NSW in both periods occurred at an unknown or already advanced stage (Additional file 2: Table S2), by which point effective treatment options are limited [33]. Despite Australia having the highest incidence of melanoma worldwide [5] we found no significant variation in survival by SES in NSW, which is consistent with previous findings [34]. This finding is likely associated with the time–delayed effects of long running and effective skin cancer awareness campaigns in Australia, which have developed a strong culture of protective behaviours [35, 36]. Patient ethnicity has been associated with both melanoma incidence and survival internationally [37, 38] though this data is not recorded by the registry and so any potential confounding of survival rates by ethnicity could not be controlled for in our analysis. Australian evidence of this association is both limited and inconclusive [39]. Data on anatomic location of melanomas was not included in this study, but previous Australian studies reported that melanomas most commonly occurred on the trunk and limbs, areas which have relatively higher survival rates [40], and that anatomic location of melanomas did not vary significantly by SES [34, 41].

We found significant differences in the distribution of stage at diagnosis between SES groups, with low SES patients more often presenting at more advanced or unknown stage for several cancers (online Additional file 2: Table S2) as reported previously [42]. This is consistent with evidence of lower screening participation among more disadvantaged groups in Australia [43] and internationally [44]. However this stage differential by SES is unlikely to explain the survival differential observed in this study, because adjusting for spread of cancer did not greatly alter our estimates. While some misclassification of recorded stage information by the Registry has been reported [45, 46], our findings suggest that increasing early diagnosis of cancers is less important than improving non-diagnostic factors, such as patient lifestyle and treatment factors, in reducing survival disparities in NSW. Notable exceptions to this were cervical and prostate cancers, which both had significant survival differentials over time prior to stage adjustment that became insignificant after adjustment. Consequently, socioeconomic variation in rates of early diagnosis may be a possible contributor to disparities in cervical and prostate cancer survival.

Patient lifestyle factors may impact on cancer survival by affecting overall health. Australian and international reports have shown that lower socioeconomic groups had significantly higher occurrences of poor lifestyle behaviours [47, 48]. Some lifestyle factors such as smoking [48] and comorbidities [49] have been shown to directly impact on the benefits of cancer treatment. However, a recent population-based study in the US found that socioeconomic disparities in breast cancer survival continued after controlling for several comorbid conditions [50], suggesting that variations in comorbidity cannot fully explain survival disparities. While we did not specifically adjust for patient comorbid conditions in our study, we did use SES-specific life tables for relative survival calculations to reduce the effect on mortality from different levels of competing causes of death across the population.

Variation in cancer management by SES may also contribute to disparities in survival, as patients of lower SES are more likely to receive sub-optimal or non-guideline therapy [21, 28]. Reduced compliance with recommended treatment regimes in low SES patients may also contribute to lower survival rates [51]. Australia’s universal healthcare system should provide consistent access to cancer treatments to all socioeconomic groups. However, it has been suggested that poorer survival in patients from lower socioeconomic areas in Australia is affected more by health system features, such as unequal access to specialist treatment centres across NSW [52]. We did not have access to information on treatment or patient management in this study, so we were unable to investigate these suggestions further.

The number of lives that might be extended beyond 5 years from diagnosis has been used previously to highlight the importance of socioeconomic survival disparities and demonstrate the potential public health benefits of improving cancer services [6, 17, 20, 53]. Estimating the number of these “avoidable deaths” (or lives “potentially saved”) can assist health authorities in allocating cancer services and resources to areas of greatest need, and increase attention on the need to further explore causes of socioeconomic variation in survival [17]. The increased number of reported avoidable deaths over time reflects both the higher incidence and improved cancer survival in NSW. The observed increases in the percentage of total excess deaths that are avoidable emphasises the trend of persistent cancer survival disparities between socioeconomic groups in NSW. These results indicate that the greatest benefit would be derived from reducing survival disparities for lung cancer patients, and that focused health and social policies should be implemented to address these disparities, as suggested previously [6]. Additional benefit would also be achieved by reducing disparities in colorectal cancer survival.

## Conclusions

In conclusion, we have reported that survival disparities by area-level SES have persisted over time for several cancers in NSW after adjusting for stage at diagnosis. While the causes of these socioeconomic disparities in survival are not thoroughly understood, variations in treatment, patient characteristics and health system factors may contribute. Despite increased awareness of SES disparities in cancer survival, and overall increases in cancer survival, this study suggests that recent health and social policies in NSW have not been effective in reducing socioeconomic inequalities in survival.

## Ethics statement

This study involves analysis of routinely collected data and the records were de-identified (name, address, date of birth had been removed) before being provided to the research team. As a large proportion of the individuals would likely have moved or died since their diagnosis of cancer, which could have been more than 20 years ago, it would have been impracticable to seek consent, and thus the NSW Population and Health Service Research Ethics Committee waived the conditions for consent and approved the study (reference #2013/06/464).

## Additional files

Additional file 1: Table S1. Number of cancer cases and distribution by socioeconomic disadvantage in New South Wales, Australia, 1996–2000 and 2004–2008. (DOCX 18 kb) Additional file 2: Table S2. Stage distribution (%) by socioeconomic status for 10 cancers in NSW, Australia, 1996–2000 and 2004–2008. (DOCX 49 kb)

## Abbreviations

SES: socioeconomic status
NSW: New South Wales
RER: relative excess risk
