# Supplementary material for: Cancer survival in New South Wales, Australia: socioeconomic disparities remain despite overall improvements
Source: BMC Cancer. 2016 Feb 1;16:48. doi: 10.1186/s12885-016-2065-z (PMC4736306; doi:10.1186/s12885-016-2065-z)
Supplement: Additional file 1: Table S1. — Number of cancer cases and distribution by socioeconomic disadvantage in New South Wales, Australia, 1996–2000 and 2004–2008. (DOCX 18 kb) [file 12885_2016_2065_MOESM1_ESM.docx]

**Additional file1**

**Table S1: Number of cancer cases and distribution by socioeconomic disadvantage in New South Wales, Australia, 1996-2000 and 2004-2008**

| Cancer | Number of Cases | | Percentage of cases by SES disadvantage quintile, early and late periods | | | | | | | | | | | | | |
| --- | --- | --- | --- | --- | --- | --- | --- | --- | --- | --- | --- | --- | --- | --- | --- | --- |
|  |  |  | Least | |  | Second | |  | Third | |  | Fourth | |  | Most | |
|  | 1996-2000 | 2004-2008 | Early | Late |  | Early | Late |  | Early | Late |  | Early | Late |  | Early | Late |
| Stomach | 3,912 | 4,711 | 20.8 | 20.2 |  | 21.6 | 20.4 |  | 19.9 | 19.7 |  | 18.4 | 19.0 |  | 19.3 | 20.6 |
| Colorectum | 28,089 | 44,397 | 21.8 | 20.4 |  | 22.6 | 21.4 |  | 19.4 | 19.6 |  | 16.4 | 17.6 |  | 19.7 | 21.0 |
| Liver | 1,160 | 2,312 | 25.3 | 22.0 |  | 21.5 | 20.3 |  | 15.3 | 16.9 |  | 19.3 | 20.8 |  | 18.6 | 20.0 |
| Lung | 15,074 | 18,868 | 18.6 | 17.0 |  | 20.5 | 19.7 |  | 20.0 | 20.2 |  | 19.2 | 20.3 |  | 21.8 | 22.9 |
| Melanoma | 22,293 | 42,347 | 20.3 | 20.2 |  | 23.4 | 22.5 |  | 19.8 | 20.4 |  | 16.4 | 16.8 |  | 20.1 | 20.1 |
| Breast | 29,869 | 53,657 | 24.5 | 23.9 |  | 22.1 | 21.7 |  | 18.7 | 18.7 |  | 16.5 | 17.1 |  | 18.3 | 18.6 |
| Cervix | 2,723 | 3,745 | 21.0 | 20.1 |  | 20.5 | 20.8 |  | 19.4 | 19.6 |  | 17.2 | 17.3 |  | 21.9 | 22.1 |
| Uterus | 3,622 | 6,560 | 22.0 | 21.0 |  | 22.7 | 21.1 |  | 19.0 | 19.4 |  | 17.3 | 18.7 |  | 19.0 | 19.9 |
| Ovary | 2,662 | 3,839 | 23.3 | 23.7 |  | 23.4 | 21.9 |  | 18.0 | 18.1 |  | 16.4 | 16.5 |  | 18.9 | 19.7 |
| Prostate | 29,830 | 57,823 | 21.7 | 21.0 |  | 22.1 | 21.5 |  | 19.8 | 19.8 |  | 16.4 | 17.4 |  | 20.0 | 20.3 |

* Early period: 1996-2000. Late period: 2004-2008.
