# Supplementary material for: Cancer survival in New South Wales, Australia: socioeconomic disparities remain despite overall improvements
Source: BMC Cancer. 2016 Feb 1;16:48. doi: 10.1186/s12885-016-2065-z (PMC4736306; doi:10.1186/s12885-016-2065-z)
Supplement: Additional file 2: Table S2. — Stage distribution (%) by socioeconomic status for 10 cancers in NSW, Australia, 1996–2000 and 2004–2008. (DOCX 49 kb) [file 12885_2016_2065_MOESM2_ESM.docx]

**Additional file 2**

**Table S2: Stage distribution (%) by socioeconomic status for 10 cancers in NSW, Australia, 1996-2000 and 2004-2008**

| Cancer | Stage distribution by SES for 10 Cancers (Row %) | | | | | | | | | | | |
| --- | --- | --- | --- | --- | --- | --- | --- | --- | --- | --- | --- | --- |
|  | 1996-2000 | | | | | |  | 2004-2008 | | | | |
|  |  | **Localised** | **Regional** | **Distant** | **Unknown** | **Total** |  | **Localised** | **Regional** | **Distant** | **Unknown** | **Total** |
| Stomach |  | 936(23.9) | 1,562(39.9) | 739(18.9) | 675(17.3) | 3,912 |  | 1,448(30.7) | 1,636(34.7) | 984(20.9) | 643(13.6) | 4,711 |
|  | Least | 221(27.2) | 312(38.4) | 156(19.2) | 124(15.3) | 813 |  | 315(33.1) | 308(32.4) | 201(21.1) | 127(13.4) | 951 |
|  | Second | 219(25.9) | 376(44.4) | 135(16.0) | 116(13.7) | 846 |  | 298(31.0) | 341(35.4) | 205(21.3) | 118(12.3) | 962 |
|  | Third | 173(22.2) | 294(37.8) | 154(19.8) | 157(20.2) | 778 |  | 286(30.8) | 323(34.8) | 176(18.9) | 144(15.5) | 929 |
|  | Fourth | 164(22.8) | 284(39.4) | 147(20.4) | 125(17.4) | 720 |  | 271(30.2) | 321(35.8) | 176(19.6) | 129(14.4) | 897 |
|  | Most | 159(21.1) | 296(39.2) | 147(19.5) | 153(20.3) | 755 |  | 278(28.6) | 343(35.3) | 226(23.3) | 125(12.9) | 972 |
|  |  |  |  |  |  |  |  |  |  |  |  |  |
| Colorectum |  | 8,712(31.0) | 13,028(46.4) | 3,327(11.8) | 3,022(10.8) | 28,089 |  | 16,594(37.4) | 18,898(42.6) | 4,758(10.7) | 4,147(9.3) | 44,397 |
|  | Least | 1,996(32.6) | 2,860(46.8) | 701(11.5) | 559(9.1) | 6,116 |  | 3,569(39.5) | 3,913(43.3) | 867(9.6) | 690(7.6) | 9,039 |
|  | Second | 2,012(31.7) | 2,916(45.9) | 780(12.3) | 648(10.2) | 6,356 |  | 3,589(37.8) | 4,075(43.0) | 986(10.4) | 835(8.8) | 9,485 |
|  | Third | 1,636(30.0) | 2,623(48.1) | 615(11.3) | 577(10.6) | 5,451 |  | 3,290(37.7) | 3,667(42.1) | 910(10.4) | 852(9.8) | 8,719 |
|  | Fourth | 1,410(30.5) | 2,159(46.7) | 556(12.0) | 494(10.7) | 4,619 |  | 2,768(35.4) | 3,306(42.3) | 955(12.2) | 787(10.1) | 7,816 |
|  | Most | 1,658(29.9) | 2,470(44.5) | 675(12.2) | 744(13.4) | 5,547 |  | 3,378(36.2) | 3,937(42.2) | 1,040(11.1) | 983(10.5) | 9,338 |
|  |  |  |  |  |  |  |  |  |  |  |  |  |
| Liver |  | 371(32.0) | 79(6.8) | 141(12.2) | 569(49.1) | 1,160 |  | 1,020(44.1) | 175(7.6) | 352(15.2) | 765(33.1) | 2,312 |
|  | Least | 120(40.8) | 24(8.2) | 32(10.9) | 118(40.1) | 294 |  | 232(45.7) | 42(8.3) | 79(15.6) | 155(30.5) | 508 |
|  | Second | 64(25.7) | 20(8.0) | 34(13.7) | 131(52.6) | 249 |  | 203(43.3) | 36(7.7) | 75(16.0) | 155(33.0) | 469 |
|  | Third | 48(27.1) | 12(6.8) | 23(13.0) | 94(53.1) | 177 |  | 184(47.1) | 32(8.2) | 57(14.6) | 118(30.2) | 391 |
|  | Fourth | 71(31.7) | 13(5.8) | 25(11.2) | 115(51.3) | 224 |  | 220(45.7) | 29(6.0) | 75(15.6) | 157(32.6) | 481 |
|  | Most | 68(31.5) | 10(4.6) | 27(12.5) | 111(51.4) | 216 |  | 181(39.1) | 36(7.8) | 66(14.3) | 180(38.9) | 463 |
|  |  |  |  |  |  |  |  |  |  |  |  |  |
| Lung |  | 3,293(21.8) | 3,185(21.1) | 4,218(28.0) | 4,378(29.0) | 15,074 |  | 5,024(26.6) | 3,541(18.8) | 6,162(32.7) | 4,141(21.9) | 18,868 |
|  | Least | 622(22.1) | 675(24.0) | 857(30.5) | 655(23.3) | 2,809 |  | 892(27.9) | 614(19.2) | 1,116(34.9) | 579(18.1) | 3,201 |
|  | Second | 738(23.9) | 701(22.7) | 849(27.5) | 796(25.8) | 3,084 |  | 1,002(27.0) | 674(18.2) | 1,200(32.4) | 833(22.5) | 3,709 |
|  | Third | 648(21.5) | 549(18.2) | 795(26.4) | 1,020(33.9) | 3,012 |  | 990(26.0) | 724(19.0) | 1,216(32.0) | 875(23.0) | 3,805 |
|  | Fourth | 638(22.1) | 594(20.6) | 808(28.0) | 850(29.4) | 2,890 |  | 1,005(26.2) | 719(18.7) | 1,192(31.1) | 920(24.0) | 3,836 |
|  | Most | 647(19.7) | 666(20.3) | 909(27.7) | 1,057(32.2) | 3,279 |  | 1,135(26.3) | 810(18.8) | 1,438(33.3) | 934(21.6) | 4,317 |
|  |  |  |  |  |  |  |  |  |  |  |  |  |
| Melanoma |  | 19,872(89.1) | 849(3.8) | 771(3.5) | 801(3.6) | 22,293 |  | 37,146(87.7) | 2,137(5.0) | 1,265(3.0) | 1,799(4.2) | 42,347 |
|  | Least | 4,074(89.9) | 152(3.4) | 151(3.3) | 155(3.4) | 4,532 |  | 7,530(88.0) | 411(4.8) | 250(2.9) | 366(4.3) | 8,557 |
|  | Second | 4,627(88.9) | 193(3.7) | 195(3.7) | 191(3.7) | 5,206 |  | 8,406(88.4) | 460(4.8) | 275(2.9) | 366(3.8) | 9,507 |
|  | Third | 3,978(90.0) | 171(3.9) | 130(2.9) | 142(3.2) | 4,421 |  | 7,575(87.7) | 432(5.0) | 274(3.2) | 353(4.1) | 8,634 |
|  | Fourth | 3,231(88.6) | 149(4.1) | 142(3.9) | 124(3.4) | 3,646 |  | 6,194(86.9) | 405(5.7) | 214(3.0) | 315(4.4) | 7,128 |
|  | Most | 3,962(88.3) | 184(4.1) | 153(3.4) | 189(4.2) | 4,488 |  | 7,441(87.3) | 429(5.0) | 252(3.0) | 399(4.7) | 8,521 |
|  |  |  |  |  |  |  |  |  |  |  |  |  |
| Breast |  | 16,111(53.9) | 9,480(31.7) | 965(3.2) | 3,313(11.1) | 29,869 |  | 29,862(55.7) | 17,813(33.2) | 1,809(3.4) | 4,173(7.8) | 53,657 |
|  | Least | 4,152(56.8) | 2,183(29.9) | 224(3.1) | 749(10.2) | 7,308 |  | 7,357(57.4) | 4,167(32.5) | 360(2.8) | 924(7.2) | 12,808 |
|  | Second | 3,560(53.9) | 2,146(32.5) | 200(3.0) | 693(10.5) | 6,599 |  | 6,497(55.7) | 3,954(33.9) | 373(3.2) | 844(7.2) | 11,668 |
|  | Third | 2,962(53.2) | 1,822(32.7) | 171(3.1) | 617(11.1) | 5,572 |  | 5,591(55.7) | 3,338(33.3) | 322(3.2) | 786(7.8) | 10,037 |
|  | Fourth | 2,600(52.8) | 1,604(32.6) | 157(3.2) | 561(11.4) | 4,922 |  | 4,953(54.0) | 3,127(34.1) | 332(3.6) | 759(8.3) | 9,171 |
|  | Most | 2,837(51.9) | 1,725(31.5) | 213(3.9) | 693(12.7) | 5,468 |  | 5,464(54.8) | 3,227(32.4) | 422(4.2) | 860(8.6) | 9,973 |
|  |  |  |  |  |  |  |  |  |  |  |  |  |
| Cervix |  | 1,492(54.8) | 594(21.8) | 112(4.1) | 525(19.3) | 2,723 |  | 2,128(56.8) | 793(21.2) | 162(4.3) | 662(17.7) | 3,745 |
|  | Least | 306(53.6) | 153(26.8) | 27(4.7) | 85(14.9) | 571 |  | 434(57.6) | 177(23.5) | 26(3.5) | 116(15.4) | 753 |
|  | Second | 296(53.0) | 125(22.4) | 21(3.8) | 117(20.9) | 559 |  | 435(55.8) | 165(21.2) | 39(5.0) | 141(18.1) | 780 |
|  | Third | 289(54.7) | 104(19.7) | 21(4.0) | 114(21.6) | 528 |  | 420(57.1) | 141(19.2) | 27(3.7) | 147(20.0) | 735 |
|  | Fourth | 270(57.6) | 88(18.8) | 24(5.1) | 87(18.6) | 469 |  | 376(57.9) | 134(20.6) | 25(3.9) | 114(17.6) | 649 |
|  | Most | 331(55.5) | 124(20.8) | 19(3.2) | 122(20.5) | 596 |  | 463(55.9) | 176(21.3) | 45(5.4) | 144(17.4) | 828 |
|  |  |  |  |  |  |  |  |  |  |  |  |  |
| Uterus |  | 2,289(63.2) | 578(16.0) | 233(6.4) | 522(14.4) | 3,622 |  | 4,338(66.1) | 1,101(16.8) | 405(6.2) | 716(10.9) | 6,560 |
|  | Least | 515(64.6) | 138(17.3) | 61(7.7) | 83(10.4) | 797 |  | 961(69.8) | 222(16.1) | 86(6.3) | 107(7.8) | 1,376 |
|  | Second | 527(64.2) | 130(15.8) | 50(6.1) | 114(13.9) | 821 |  | 899(65.1) | 234(16.9) | 104(7.5) | 145(10.5) | 1,382 |
|  | Third | 430(62.5) | 116(16.9) | 38(5.5) | 104(15.1) | 688 |  | 842(66.1) | 216(17.0) | 66(5.2) | 149(11.7) | 1,273 |
|  | Fourth | 395(62.9) | 101(16.1) | 31(4.9) | 101(16.1) | 628 |  | 783(64.0) | 214(17.5) | 80(6.5) | 147(12.0) | 1,224 |
|  | Most | 422(61.3) | 93(13.5) | 53(7.7) | 120(17.4) | 688 |  | 853(65.4) | 215(16.5) | 69(5.3) | 168(12.9) | 1,305 |
|  |  |  |  |  |  |  |  |  |  |  |  |  |
| Ovary |  | 571(21.5) | 418(15.7) | 1,329(49.9) | 344(12.9) | 2,662 |  | 1,053(27.4) | 622(16.2) | 1,749(45.6) | 415(10.8) | 3,839 |
|  | Least | 151(24.3) | 111(17.9) | 291(46.9) | 68(11.0) | 621 |  | 276(30.3) | 155(17.0) | 388(42.6) | 92(10.1) | 911 |
|  | Second | 142(22.8) | 93(15.0) | 309(49.7) | 78(12.5) | 622 |  | 239(28.5) | 136(16.2) | 386(46.0) | 79(9.4) | 840 |
|  | Third | 105(21.9) | 70(14.6) | 240(50.1) | 64(13.4) | 479 |  | 194(27.9) | 127(18.2) | 300(43.1) | 75(10.8) | 696 |
|  | Fourth | 73(16.7) | 71(16.2) | 230(52.6) | 63(14.4) | 437 |  | 153(24.1) | 81(12.8) | 319(50.3) | 81(12.8) | 634 |
|  | Most | 100(19.9) | 73(14.5) | 259(51.5) | 71(14.1) | 503 |  | 191(25.2) | 123(16.2) | 356(47.0) | 88(11.6) | 758 |
|  |  |  |  |  |  |  |  |  |  |  |  |  |
| Prostate |  | 12,478(41.8) | 1,445(4.8) | 1,329(4.5) | 14,578(48.9) | 29,830 |  | 28,865(49.9) | 3,388(5.9) | 1,377(2.4) | 24,193(41.8) | 57,823 |
|  | Least | 3,123(48.2) | 415(6.4) | 263(4.1) | 2,674(41.3) | 6,475 |  | 6,873(56.5) | 887(7.3) | 249(2.0) | 4,162(34.2) | 12,171 |
|  | Second | 2,953(44.8) | 302(4.6) | 299(4.5) | 3,044(46.1) | 6,598 |  | 6,572(52.9) | 813(6.5) | 275(2.2) | 4,770(38.4) | 12,430 |
|  | Third | 2,234(37.8) | 267(4.5) | 230(3.9) | 3,172(53.7) | 5,903 |  | 5,327(46.5) | 566(4.9) | 258(2.3) | 5,304(46.3) | 11,455 |
|  | Fourth | 1,908(39.1) | 198(4.1) | 219(4.5) | 2,558(52.4) | 4,883 |  | 4,647(46.2) | 467(4.6) | 244(2.4) | 4,695(46.7) | 10,053 |
|  | Most | 2,260(37.8) | 263(4.4) | 318(5.3) | 3,130(52.4) | 5,971 |  | 5,446(46.5) | 655(5.6) | 351(3.0) | 5,262(44.9) | 11,714 |
|  |  |  |  |  |  |  |  |  |  |  |  |  |

Localised = SEER Stage I; Regional = SEER Stages II-III; Distant = SEER Stage IV; Unknown = no/missing data.
